# A user-friendly nomogram for predicting radioiodine refractory differentiated thyroid cancer

**DOI:** 10.3389/fendo.2023.1109439

**Published:** 2023-02-10

**Authors:** Chao Meng, Juanjuan Song, Wen Long, Zhuanzhuan Mu, Yuqing Sun, Jun Liang, Yansong Lin

**Affiliations:** ^1^Department of Oncology, Key Laboratory of Carcinogenesis and Translational Research (Ministry of Education/Beijing), Peking University Cancer Hospital & Institute, Beijing, China; ^2^Department of Nuclear Medicine, State Key Laboratory of Complex Severe and Rare Diseases, Peking Union Medical College (PUMC) Hospital, Chinese Academy of Medical Sciences & PUMC, Beijing, China; ^3^Beijing Key Laboratory of Molecular Targeted Diagnosis and Therapy in Nuclear Medicine, Beijing, China; ^4^Department of Oncology, Peking University International Hospital, Beijing, China; ^5^Department of Nuclear Medicine, Peking University International Hospital, Beijing, China; ^6^Department of Nuclear Medicine, State Key Laboratory of Oncology in South China, Sun Yat-sen University Cancer Center, Guangzhou, China

**Keywords:** neoplasm metastasis, thyroid cancer, thyroglobulin, radioiodine refractory, nomogram

## Abstract

**Background:**

The diagnosis of radioiodine refractory differentiated thyroid cancer (RAIR-DTC) is primarily based on clinical evolution and iodine uptake over the lesions, which is still time-consuming, thus urging a predictive model for timely RAIR-DTC informing. The aim of this study was to develop a nomogram model for RAIR prediction among DTC patients with distant metastases (DM).

**Methods:**

Data were extracted from the treatment and follow-up databases of Peking Union Medical College Hospital between 2010 and 2021. A total of 124 patients were included and divided into RAIR (n=71) and non-RAIR (n=53) according to 2015 ATA guidelines. All patients underwent total thyroidectomy followed by at least two courses of RAI treatment. Serological markers and various clinical, pathological, genetic status, and imaging factors were integrated into this study. The pre-treatment stimulated Tg and pre- and post-treatment suppressed Tg at the first and second course RAI treatment were defined as s-Tg1, s-Tg2, sup-Tg1, and sup-Tg2, respectively. Δs-Tg denoted s-Tg1/s-Tg2, and Δs-TSH denoted s-TSH1/s-TSH2. Multivariate logistic regression and correlation analysis were utilized to determine the independent predictors of RAIR. The performance of the nomogram was assessed by internal validation and receiver operating characteristic (ROC) curve, and benefit in clinical decision-making was assessed using decision curve.

**Results:**

In univariate logistic regression, nine possible risk factors were related to RAIR. Correlation analysis showed four of the above factors associated with RAIR. Through multivariate logistic regression, Δs-Tg/Δs-TSH<1.50 and age upon diagnosis were obtained to develop a convenient nomogram model for predicting RAIR. The model was internally validated and had good predictive efficacy with an AUC of 0.830, specificity of 0.830, and sensitivity of 0.755. The decision curve also showed that if the model is used for clinical decision-making when the probability threshold is between 0.23 and 0.97, the net benefit of patients is markedly higher than that of the TreatAll and TreatNone control groups.

By using 1.50 as a cut-off ofΔs-Tg/Δs-TSH, differing biochemical progression among the generally so-called RAIR can be further stratified as meaningfully rapidly or slowly progressive patients (*P*=0.012).

**Conclusions:**

A convenient user-friendly nomogram model was developed with good predictive efficacy for RAIR. The progression of RAIR can be further stratified as rapidly or slowly progressive by using 1.50 as a cut-off value of Δs-Tg/Δs-TSH.

## Introduction

The incidence of thyroid cancer has kept rising globally (1). Differentiated thyroid cancer (DTC)accounts for more than 90% of thyroid cancers and consists mainly of papillary thyroid cancer (PTC) and follicular thyroid cancer (FTC) (2, 3). Most DTCs carry a favorable prognosis after thyroidectomy, selective radioactive iodine (RAI) treatment, and thyroid stimulating hormone (TSH) -suppressive treatment. However, roughly 7-23% of DTC patients develop distant metastases (DM) (4–6). Among these patients, two-thirds become radioiodine refractory DTC (RAIR-DTC) and have a much lower 10-year survival rate than those RAI responsive patients (10 vs. 56%), which is the most common cause of cancer-related deaths, and pose a great challenge in the management of DTC (7–9). Given the lack of benefit of repeated RAI treatment to these patients and the increasing risk of side effects, timely identifying the RAIR-DTC is imperative for such patients to avoid unnecessary RAI treatment and gain more time for the effective treatment regimen like Multi-Kinase Inhibitors (MKIs).

The definition of RAIR has been outlined in the 2015 ATA guidelines (10), which are mainly based on imaging manifestation characterized by the loss of radioiodine uptake and increasing level of the tumor marker thyroglobulin (Tg). Of note, the diagnosis of RAIR-DTC is primarily based on clinical evolution and iodine uptake characteristics rather than pathological characteristics. While controversies existed particularly in terms of the predictive value of ^131^I whole body scan (^131^I-WBS) imaging and imaging heterogeneity among multiple metastases (11). Thus the ultimate identification still requires long-term follow-up after RAI treatment, which is time-consuming and often induces a delayed judgment that deprives access to timely and effective therapeutic approaches.

Over recent years, clinicopathological characteristics, as well as molecular features, have been recognized as meaningful indicators that could be utilized for RAIR prediction. And factors like older age, larger primary tumor size, extrathyroidal extension (ETE), BRAF^V600E^ mutation, TERT promoter mutation, and high-risk histological subtypes were reported to be correlated with RAIR-DTC respectively, according to different retrospective studies, with an unfavorable median overall survival (OS) (12–14). While factors identified in each study alone still showed poor performance in predicting individual risk of RAIR, thus a predictive model is needed to integrate multiple factors as a whole. The nomogram is an effective model that takes the weights of relevant factors into consideration and integrates the independent factors for predicting the risk probability of a special clinical event, which is valuable for clinical decision-making and risk stratification (15, 16). Only two studies have reported the nomogram model to predict RAIR-DTC so far to our knowledge, which mainly took static clinicopathologic and costly ^18^F-FDG PET/CT molecular imaging features into account, while both of which lacked dynamic assessment and were not universally available due to economic burden (17, 18). While Tg as the most critical real-time tumor marker was ignored, which has an irreplaceable role in the follow-up and management of thyroid cancer after surgery to predict recurrence and metastasis and assess long-term survival (19–22).

This study aims to develop a prediction nomogram model of RAIR by integrating serological markers such as Tg, various clinical, pathological, genetic status, and imaging factors for predicting RAIR among patients with DM-DTC.

## Patients and methods

The study protocol was approved by the ethical board of the Chinese Academy of Medical Sciences and Peking Union Medical College Hospital (PUMCH).

### Patients

In this retrospective study, we extracted data for all DM-DTC patients (n=369) from 2205 thyroid cancer patients treated with total or near-total thyroidectomy followed by RAI treatment between 2010 and 2021, with follow-up through November 1, 2021. Patients with DM-DTC were defined with any of the following: ① metastatic lesions confirmed by pathology, ② focal or diffuse uptake in metastatic lesions on ^131^I-WBS after excluding the contamination and physiological RAI uptake, with or without positive findings on other complementary imaging modalities (chest CT, x-rays, MRI, bone scintigraphy or ^18^F-FDG PET/CT) or elevated Tg levels, ③ positive findings on ^18^F-FDG PET/CT after excluding other malignancies and benign diseases, with a rising Tg level, despite of the negative ^131^I-WBS results, ④ negative findings on functional imaging, but structural lesions suggested by other imaging instruments with a rising Tg level after excluding other malignancies and benign diseases. Patients’ exclusion criteria with either of the following: ① no distant metastatic lesions identified on imaging, ② absence of serial serological data, ③ absence of information on the first two rounds of RAI treatment, ④ absence of follow-up information.

**Figure 1:**
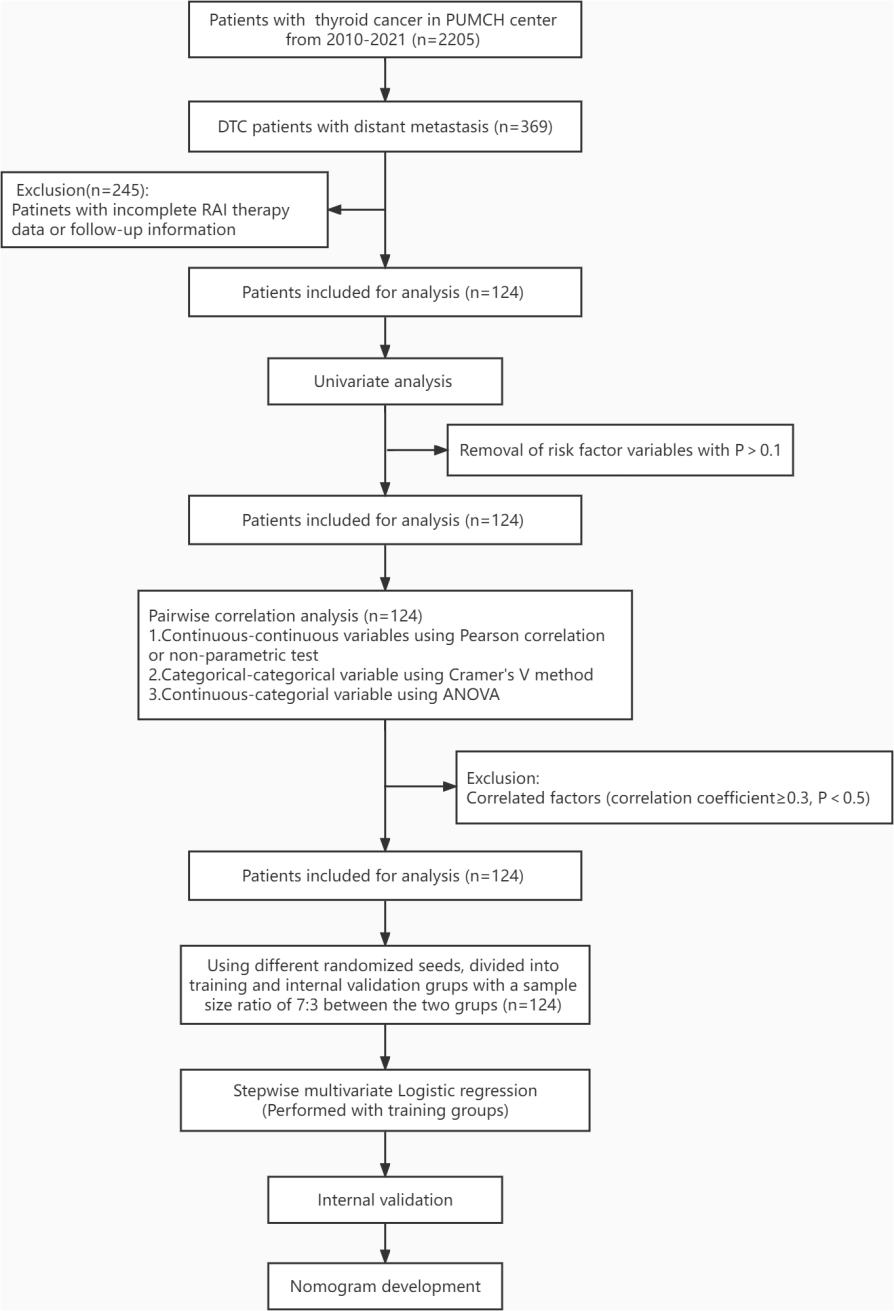
Workflow for patient selection and development of the nomogram model to predict RAIR among DM-DTC patients. PUMCH, Peking Union Medical College Hospital; RAIR, radioiodine refractory; DM, distant metastases; DTC, differentiated thyroid cancer.

RAIR definition was based on the 2015 ATA guidelines: (i) the malignant/metastatic tissue does not ever concentrate RAI (no uptake outside the thyroid bed at the first therapeutic WBS), (ii) the tumor tissue loses the ability to concentrate RAI after previous evidence of RAI-avid disease (in the absence of stable iodine contamination), (iii) RAI is concentrated in some lesions but not in others; and (iv) metastatic disease progresses despite significant concentration of RAI (10). A total of 124 DM-DTC patients were finally enrolled (**Figure 1**). The overall median follow-up was 51.5 months (interquartile range:32.75,70.00 months). Patients were further divided into non-RAIR and RAIR.

### Postoperative RAI treatment

A serum thyroid stimulating hormone (TSH) level higher than 30mU/L was achieved by thyroid hormone withdrawal (THW) before RAI treatment. All patients were instructed with a low iodine diet from the beginning of THW to 3 weeks after RAI treatment. Patients were administrated with a range of 3.7–7.4GBq (100-200mCi) RAI treatment dose. Repeated RAI treatment was usually performed 6 to 12 months after the previous treatment.

Stimulated or suppressed Tg (s-Tg or sup-Tg), TSH, and thyroglobulin antibody (TgAb) were usually tested 1 day before and 2–3 months after RAI treatment. S-Tg was defined as Tg measured after THW with a TSH level>30 mU/L. The s-Tg and TSH on the day of the first (s-Tg1, s-TSH1) and second (s-Tg2, s-TSH2) course of RAI treatment and the suppressed Tg at 1 to 3 months before (sup-Tg1) and 3 months after (sup-Tg2) the second RAI treatment were further analyzed to detect the prediction value. Post-therapeutic whole-body scan (Rx-WBS) was performed 5–7 days after RAI treatment. CT without contrast was carried out as needed. All patients were under TSH suppressive treatment using sodium levothyroxine to achieve a TSH level<0.1μIU/mL during courses of RAI treatment and subsequent follow-up.

### Measurement of Tg, TgAb and TSH

Tg and TgAb levels were determined by electrochemiluminescence immunoassay (Roche Diagnostics GmbH, Mannheim, Germany) with a functional sensitivity of 0.100 ng/mL and 10 IU/mL, respectively. TSH was determined by chemiluminescence immunoassay (Siemens Healthcare Diagnostics Inc, New York, New York, USA), with a measuring range from 0.004 to 150μIU/mL. TgAb values>115 IU/mL were considered positive. Rx-WBS was obtained in the anterior and posterior projections using dual-head gamma cameras (Infinia Hawkeye; GE, Fairfield, Connecticut, USA) equipped with high-energy parallel-hole collimators, and a 20% energy window was centered at 364 keV, at a table speed of 20 cm/min for a total time of 15 min (256×1024 matrix).

### Biochemical progression

The sup-Tg level in all samples with TSH level<0.1μIU/mL and TgAb negativity (≤115IU/mL) was examined. Biochemical progression was defined as>20% increase in sup-Tg level at follow-up compared with that after thyroid remnant ablation (23).

### Gene analysis

Genomic DNA was extracted from either primary or lymph-node metastatic DTC tumors, which were sliced into 5μm-thick sections, fixed with formalin and embedded in paraffin. BRAF and TERT mutations were analyzed by real-time fluorescent quantitative polymerase chain reaction (PCR). Exon 15 of the BRAF gene containing the site for the T1799A (V600E) mutation was amplified using primers 5'- TGCTTGCTCTGA TAGGAAAA TG-3'(sense) and 5'-AGCCTCAA TTCTTACCA TCCA-3'(antisense). The TERT promoter includes two mutation hotspots: C228T and C250T. A 193-bp fragment of the TERT promoter was amplified by PCR using approximately 100 ng of genomic DNA and primers 5'-CACCCGTCCTGCCCCTTCACCTT-3'(sense) and 5'-GGCTTCCCACGTGCGCAGCAGGA-3'(antisense). The real-time fluorescent quantitative PCR protocol comprised an initial denaturation for 5 min at 95°C, 15 cycles of template enrichment (denaturation for 25 s at 95°C, annealing for 20 s at 64°C, and extension for 20 s at 72°C), and 31 amplification cycles (denaturation for 25 s at 93°C, annealing for 35 s at 60°C, and amplification cycles (denaturation for 25 s at 93°C, annealing for 35 s at 60°C, and DNA Analyzer.

### Data processing and statistical analysis

All factors included in the analysis were divided into categorical and continuous variables, with no special processing for the former. Continuous variable were handled as follows: ① remove outliers: Data less than the 1st quartile minus1.5 times the quartile difference were replaced with the 5% quantile and data greater than the 3rd quartile plus 1.5 times the quartile difference were replaced with the 95% quantile; ② remove the Tg for TgAb>115 IU/ml; ③ ROC curves were plotted for each continuous variable against the RAIR status, and variables with AUC value>0.7 were then transformed to corresponding categorical variables. A dataset was then derived after removing highly subjective variables such as TSH levels, cumulative dose, and cumulative number of RAI treatment. Very incomplete variables were removed to get the final dataset. Univariate and multivariate logistic regression was then applied to this dataset. **Figure 1** shows the workflow chart for patient selection and model construction.

Statistical analysis was performed using the R software (version 4.1.2). The packages in R that were used in this study are reported in the **Data Supplementary**. The reported statistical significance levels between groups were all two-sided, with statistical significance set at 0.05.

The Kaplan-Meier and log-rank analyses were used to assess Tg progression-free survival (Tg-PFS).

## Results

### Clinicopathologic characteristics of patients

Of 124 patients included in the analysis, 118 (95.2%) had PTC, 5 (4.0%) had FTC and 1 (0.8%) had PTC combined with FTC. The baseline clinicopathologic characteristics of 124 DM-DTC patients, including 42 men and 82 women, with a male/female ratio of 1:1.95, are summarized in **Table 1**. With the criteria for judgment, 71 patients were identified as RAIR, 53 as non-RAIR, and one RAIR case fulfilled criteria ii and iii. Patients with RAIR were more likely to be older upon diagnosis and with more advanced T staging (*P*=0.021 and 0.015, respectively). RAIR tended to have higher capsular invasion rate and larger tumor size (*P*=0.059 and 0.147, respectively). There were no statistical differences in terms of gender, tumor lesion, multifocality, and N staging.

**Table 1:** Clinicopathologic Characteristics of 124 Patients with DM-DTC.

Characteristics	Total (N=124)	Non-RAIR (N=53)	RAIR (N=71)	*P*
Age upon diagnosis (year) [mean (SD)]	35.10 (15.60)	31.38 (12.59)	37.89 (17.08)	0.021
Gender [n (%)]				0.578
Mela	42 (33.90)	16 (30.20)	26 (36.6)	
Female	82 (66.10)	37 (69.80)	45 (63.40)	
Tumor largest dimension (cm) [mean (SD)]	2.58 (1.72)	2.31 (1.59)	2.80 (1.80)	0.147
Cervical lymph node dissection [n (%)]				0.418
Yes	117 (94.40)	52 (98.10)	65 (91.50)	
No	7 (5.60)	1 (1.90)	6 (8.50)	
Pathological subtype [n (%)]				0.098
Classic PTC	29 (23.40)	15 (28.30)	14 (19.70)	
Follicular-variant PTC	36 (29.00)	19 (35.80)	17 (23.90)	
Other subtypes	21 (16.90)	6 (11.30)	15 (21.10)	
Unavailable	38 (30.60)	13 (24.50)	25 (35.20)	
Tumor lesion [n (%)]				0.441
Unilateral	59 (47.60)	28 (52.80)	31 (43.70)	
Bilateral	64 (51.60)	25 (47.20)	39 (54.90)	
Unavailable	1 (0.80)	0 (0.00)	1 (1.40)	
Multifocality [n (%)]				0.351
One lesion	36 (29.00)	19 (35.80)	17 (23.90)	
More than one lesion	80 (64.50)	31 (58.50)	49 (69.00)	
Unavailable	8 (6.50)	3 (5.70)	5 (7.00)	
Capsular invasion [n (%)]				0.059
Yes	102 (82.30)	40 (75.50)	62 (87.30)	
No	12 (9.70)	9 (17.00)	3 (4.20)	
Unavailable	10 (8.10)	4 (7.50)	6 (8.50)	
BRAF^V600E^ mutation [n (%)]				0.085
Yes	44 (35.50)	14 (26.40)	30 (42.30)	
No	63 (50.80)	33 (62.30)	30 (42.30)	
Unavailable	17 (13.70)	6 (11.30)	11 (15.50)	
TERT mutation [n (%)]				0.04
Yes	12 (9.70)	1 (1.90)	11 (15.50)	
No	46 (37.10)	21 (39.60)	25 (35.20)	
Unavailable	66 (53.20)	31 (58.50)	35 (49.30)	
AJCC-T Stage [n (%)]				0.015
T1	31 (25.0)	21 (39.6)	10 (14.10)	
T2	12 (9.7)	4 (7.5)	8 (11.30)	
T3	17 (13.7)	4 (7.5)	13 (18.30)	
T4	55 (44.4)	21 (39.6)	34 (47.90)	
Unavailable	9 (7.3)	3 (5.7)	6 (8.50)	
AJCC-N Stage [n (%)]				0.235
N0	3 (2.40)	1 (1.90)	2 (2.80)	
N1a	12 (9.70)	6 (11.30)	6 (8.50)	
N1b	100 (80.60)	45 (84.90)	55 (77.50)	
Unavailable	9 (7.30)	1 (1.90)	8 (11.30)	
Serological characteristics (ng/mL) [median (IQR)]				
s-Tg1	344.10 (62.74,500.0)	361.40 (93.14,500.0)	342.20 (60.34-500.0)	0.636
sup-Tg1	14.31 (4.75,77.51)	12.73 (2.76-69.0)	16.30 (5.03-80.04)	0.573
s-Tg2	189.96 (57.23, 500.6)	119.8 (38.44, 466)	250.2 (78.89, 603.6)	0.02
sup-Tg2	11.10 (3.15, 70.44)	7.73 (2.0, 42.37)	16.94 (6.2, 87.49)	0.027
Δs-Tg [median (IQR)]	1.06 (0.92, 1.71)	1.69 (1.00,3.7)	1.00 (0.75,1.27)	<0.001
Δs-Tg/Δs-TSH [median (IQR)]	1.37 (0.95, 2.68)	2.42 (1.58-4.11)	1.05 (0.79-1.4)	<0.001
Δs-Tg/Δs-TSH<1.50 [n (%)]				<0.001
Yes	69 (55.6)	11 (20.8)	58 (81.7)	
No	55 (44.4)	42 (79.2)	13 (18.3)	
Cumulative RAI dose (mCi) [median (IQR)]	340 (300, 450)	450.00 (300, 540)	300.00 (300, 450)	0.05
Number of RAI treatment [n (%)]				0.263
2	63 (50.8)	22 (41.5)	41 (57.7)	
3	35 (28.2)	16 (30.2)	19 (26.8)	
4	12 (9.7)	9 (17.0)	3 (4.2)	
>4	14 (11.3)	6 (11.3)	8 (11.3)	
RAIR according to 2015 ATA guidelines [n (%)]				
i			36 (50.7)	
ii			5 (7.04)	
iii			15 (21.13)	
iv			16 (22.54)	

DM, distant metastases; DTC, differentiated thyroid cancer; RAI, radioactive iodine; Δs-Tg, s-Tg1/s-Tg2; Δs-TSH, s-TSH1/ s-TSH2.

### Serological characteristics in RAIR and non-RAR patients

**Figure 2:**
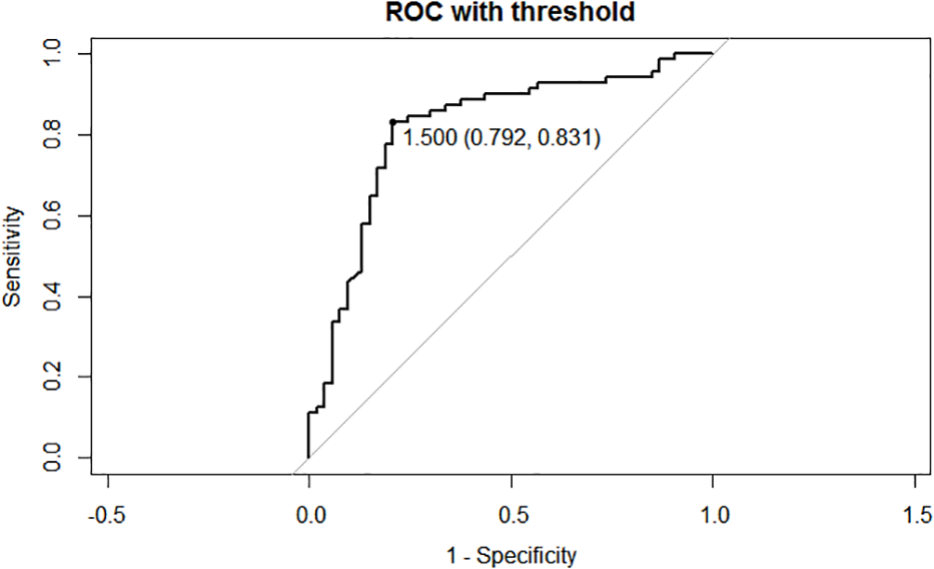
Receiver operating characteristic (ROC) curves of the cut-off of Δs-Tg/Δs-TSH. AUC: 0.818, cutoff value:1.50, sensitivity:0.831, specificity: 0.792. Δs-Tg: s-Tg1/ s-Tg2, Δs-TSH: s-TSH1/ s-TSH2, AUC: Area Under Curve.

Serological follow-up was performed on 124 patients with DM-DTC before and after the first and second RAI treatment (**Table 1**). TSH, Tg and TgAb results did not differ between the two groups before and after the first RAI treatment. Both s-Tg2 and sup-Tg2 were significantly higher in RAIR group (*P*=0.02, and 0.027, respectively). Additionally, Δs-Tg (s-Tg1/s-Tg2) was remarkably lower in RAIR than the in non-RAIR group (*P*<0.001). To minimize the influence of TSH, Δs-Tg/Δs-TSH (s-TSH1/s-TSH2) was also calculated in the analyses. Δs-Tg/Δs-TSH in RAIR was significantly lower compared to non-RAIR (*P*<0.001) and was also more evident than that of Δs-Tg. A cut-off value of Δs-Tg/Δs-TSH at 1.50 was obtained with a sensitivity of 0.831, specificity of 0.792, and AUC of 0.818, respectively (**Figure 2**).

### Results of univariate logistic regression and correlation analysis

Univariate logistic regression demonstrated that sup-TgAb1, s-Tg2, sup-Tg2, Δs-Tg, Δs-Tg/Δs-TSH, Δs-Tg/Δs-TSH<1.50, age upon diagnosis, BRAF^V600E^ mutation, and AJCC-T stage were opted risk factors for RAIR (*P*<0.1, **Table 2**), among which sup-TgAb1, sup-Tg2, BRAF^V600E^ mutation, and AJCC-T stage were removed from dataset due to data incompleteness. The remaining possible risk factors were included into the correlation analysis. Risk factors with a correlation coefficient greater than 0.3 were considered to be correlated factors. In correlation analysis, Δs-Tg was removed because of correlation with both s-Tg2 (correlation factor=-0.323) and Δs-Tg/Δs-TSH(correlation factor=0.743), leaving the following four factors to be opt-independent risk factors: s-Tg2, Δs-Tg/Δs-TSH, Δs-Tg/Δs-TSH<1.50, and age upon diagnosis.

**Table 2:** Results of univariate logistic regression.

Characteristics	*P*	Characteristics	*P*
Age upon diagnosis	0.023*	s-Tg1	0.13
Gender	0.127	sup-Tg1	0.287
Tumor largest dimension	0.153	sup-TgAb1	0.092*
Cervical lymph node dissection	0.111	s-Tg2	0.050*
Tumor lesion	0.288	sup-Tg2	0.039*
Multifocality	0.16	Δs-Tg	0.020*
BRAF^V600E^ mutation	0.037*	Δs-Tg/Δs-TSH	0.001*
AJCC-T Stage	0.083*	Δs-Tg/Δs-TSH<1.5	<0.01*
AJCC-N Stage	0.392		

*P<0.05, Δs-Tg: s-Tg1/s-Tg2, Δs-TSH: s-TSH1/ s-TSH2.

### Multivariate analysis, internal validation, and logistic model construction

**Figure 3:**
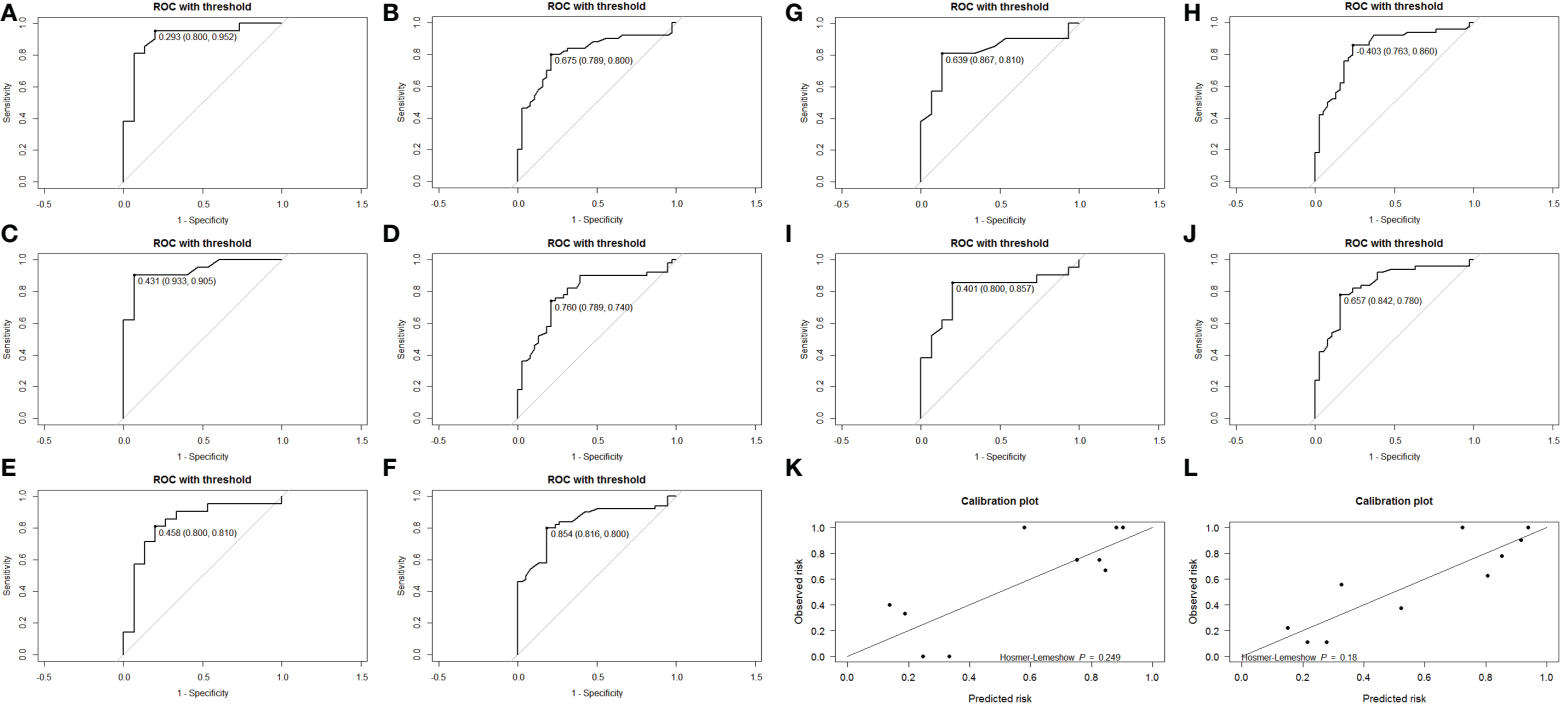
Receiver operating characteristic (ROC) curves for the internal validation set **(A, C, E, G, I)** and the training set **(B, D, F, H, J)**. Calibration plots for the last internal validation of the model, training set **(L)**, and validation set **(K)**.

**Figure 4:**
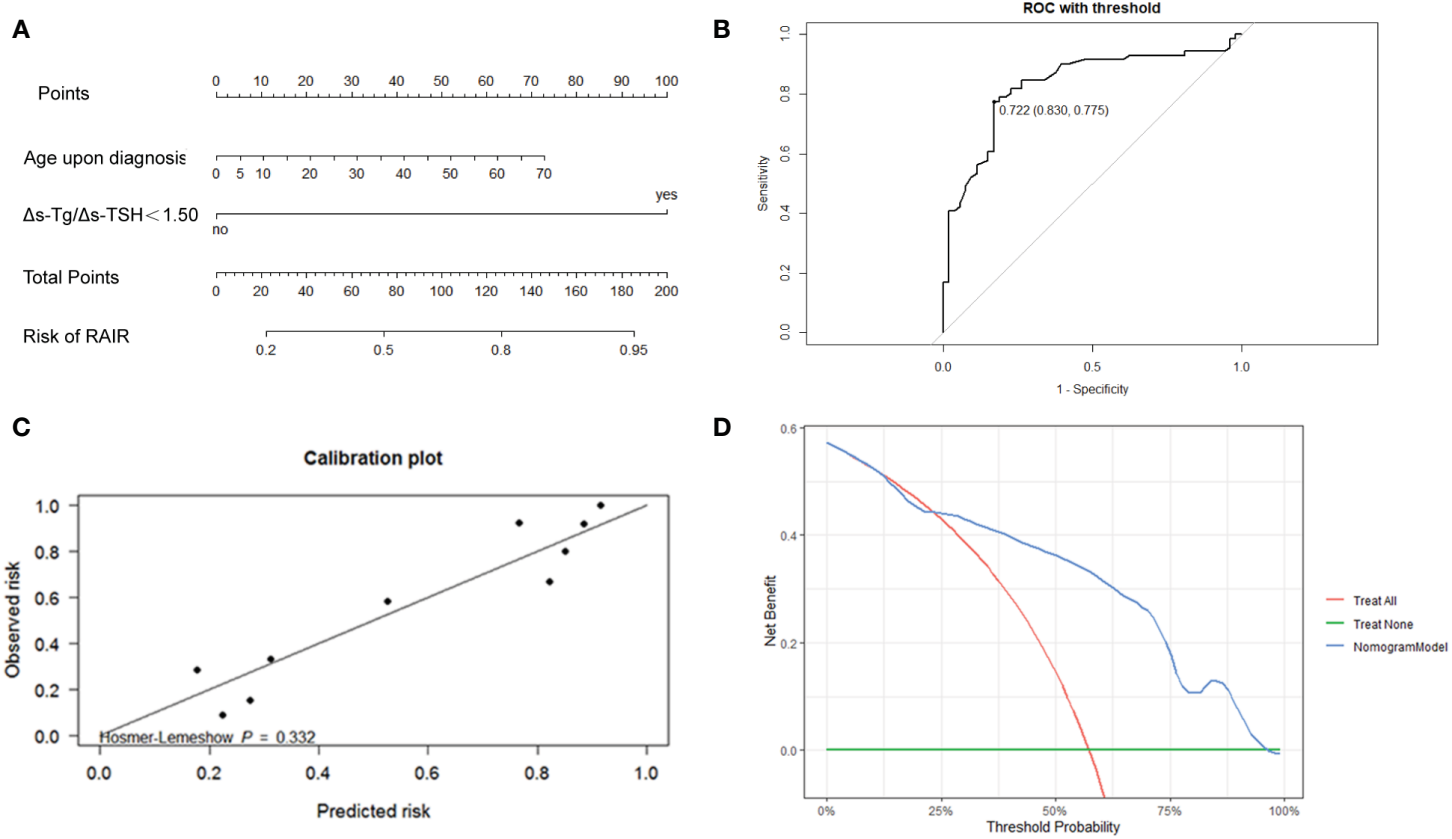
**(A)** Nomogram to develop the risk of RAIR-DTC in DTC patients with distant metastasis. **(B)** ROC curves of the nomogram model for predicting RAIR. AUC:0.830, sensitivity:0.775, specificity: 0.830. The cut-off value of risk of RAIR is 0.722. **(C)** Calibration curve. **(D)** Decision curve.

Totally, 70% of cases were randomly selected as the training set, and then stepwise multivariate logistic regression was performed using the MASS package (7.3-54) of R. The left 30% of cases were selected as the validation set. Two independent risk factors, Δs-Tg/Δs-TSH<1.50 and age upon diagnosis, were eventually screened for predicting RAIR. The above procedure was performed five times with different randomization seeds, and the results were reliable (**Figure 3**, **Table 3**). We then used the two factors (Δs-Tg/Δs-TSH<1.50 and age upon diagnosis) as independent risk factors to generate a linear Logistic regression model to predict RAIR. Based on the two independent factors obtained in stepwise multiple Logistic regression analysis, a nomogram characterized by scale line and score weight reflected to RAIR-DTC prediction was established in **Figures 4A, B**; the AUC of the model is 0.830, with a specificity of 0.830 and sensitivity of 0.755. The calibration and decision curves for our final model are also shown in **Figures 4C, D**, with the p-value for the Hosmer-Lemeshow test for the former being greater than 0.05, indicating our model’s probability of predicting RAIR is not significantly different from the actual RAIR situation. The latter shows that our model outperforms the two control groups of TreatAll and TreatNone in terms of the net benefit to patients’ clinical decision-making over a relatively significant threshold probability range of 0.24-0.97.

**Table 3:** Results of stepwise multivariate logistic regression on five random samples.

Independent risk factors	training set AUC	Internal validation set		
		AUC	Sensitivity	Specificity
Δs-Tg/Δs-TSH<1.50, Age upon diagnosis	0.810	0.913	0.952	0.800
	0.788	0.933	0.905	0.933
	0.834	0.832	0.810	0.800
	0.837	0.825	0.810	0.867
	0.842	0.805	0.857	0.800

### Impact of Δs-Tg/Δs-TSH cut-off value of 1.50 on Tg-PFS in RAIR patients

**Figure 5:**
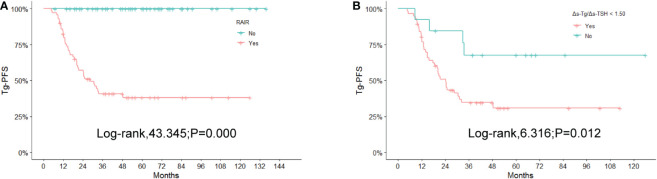
Kaplan-Meier curves for Tg-PFS **(A)** between non-RAIR and RAIR patients, **(B)** between RAIR patients with 1.50 of Δs-Tg/Δs-TSH as the cutoff value. RAIR, radioiodine refractory; Tg-PFS, thyroglobulin progression-free survival.

The results of the Kaplan-Meier and log-rank analyses of Tg-PFS in patients with RAIR are provided below. The median Tg-PFS (mTg-PFS) in 124 patients was 37 months (95% CI: 37.83-48.82 months). The mTg-PFS was 24.4 months (95% CI: 25.93-38.23 months) and 56 months (95% CI:49.91-66.88months) for RAIR and non-RAIR patients, respectively. An obviously shorter Tg-PFS was observed in RAIR than in non-RAIR (χ2 = 43.345, *P*=0.000) (**Figure 5A**).

In the RAIR group, the mTg-PFS was 20.49 months (95% CI: 21.89-33.71 months) for 58 patients with Δs-Tg/Δs-TSH<1.50 and 43.00 months (95% CI:31.58-70.76 months) for 13 patients with Δs-Tg/Δs-TSH≥1.50. The log-rank test showed a significantly shorter Tg-PFS in the former patients compared to the latter (χ2 = 6.316, P =0.012) (**Figure 5B**).

## Discussion

In the present study, we retrospectively developed and validated a nomogram model for predicting RAIR in patients with DM-DTC by integrating serological markers and various clinical, pathological, genetic status, and imaging factors. Based on univariate logistic regression, nine possible risk factors were significantly related to RAIR. And correlation analysis showed that four of the above factors displayed associations with RAIR. Two independent predictors of RAIR were finally confirmed in multivariate logistic regression analysis: Δs-Tg/Δs-TSH and age upon diagnosis, with the former parameter derived from Tg, which would be a dichotomous variable reflecting the dynamic s-Tg changing rate with the cut-off of 1.50. Internal validation was used to determine that the model had good predictive power to predict RAIR. The decision curve with net benefits was further used to assess the model’s better clinical predictive value.

Tg has been reported to be a specific biomarker produced by thyroid tissue and DTC lesions, which is well acknowledged as convenient, reproducible, and sensitive to be monitored, particularly after remnant thyroid ablation (10). It plays an essential role in the surveillance of thyroid cancer after surgery to reveal recurrence, metastasis and assess long-term survival (24). Previous researches from our team have shown that qualitative assessment of the Tg levels can help to grade the risk of recurrence and predict metastasis and RAIR in DTC patients with pulmonary metastases (20, 25). The change of Tg during TSH stimulation, such as ΔTg/ΔTSH, can be used as a specific early biochemical marker for DM-DTC (22). Wang C et al. further analyzed the percentage change of Tg before and after RAI treatment, namely ΔTg, during the first two courses in DM-DTC patients receiving multiple RAIs and showed that it was informative for RAIR (25). Based upon the above findings, Δs-Tg/Δs-TSH was applied for the first time in this study to predict RAIR. Different from the previous Tg-relevant indicators, such a parameter not only associates Tg with TSH to make it standardized and comparable but also reflects the biochemical efficacy from RAI treatment longitudinally.

Age upon diagnosis was another important independent factor manifested in this study. Age has been associated with thyroid cancer staging, RAIR prediction, and risk of death. Age above 45 and over 55 years old were significant stratification factors in the TNM staging of thyroid cancer to differentiate the risk of death, in its 7th, and 8th editions, respectively (26, 27). Other researchers have reported a significant inverse correlation between RAI avidity and different age cut-offs, such as >45, ≥46, or >55-years-old (13, 25, 28). A predictive value for OS of RAIR can be observed by using 45- and 75-years-old as cut-off, respectively (29). In a nomogram model to predict RAIR developed recently, an age cut-off of 48-years-old has also been integrated as an independent factor (18). Our finding revealed that age was also proved to be a significant predictor for RAIR as a continuous rather than a dichotomous variable in previous studies, which presumably related to the wide range of age in the entire population in our research. Thus, it allows a specific personalized nomogram scoring in patients with different ages upon diagnosis.

Thus, unlike other scoring systems in identifying RAIR with either complex or high-cost index such as ^18^F-FDG-PET (18), we developed this nomogram model with promising predictive RAIR efficacy using two readily accessible factors described above. We believe it might be a user-friendly tool in routine clinical practice, particularly in China where recombinant human TSH (rhTSH) remains unavailable currently and thyroid hormone withdrawal is the only modality for TSH stimulation, which makes rhTSH aided-diagnostic RAI whole-body scan (DxWBS) for RAIR identification impossible. And due to its economic feasibility and practicability, our nomogram model might be an alternative for some other developing countries, where rhTSH cannot be affordable for most of the patients.

**Figure 6:**
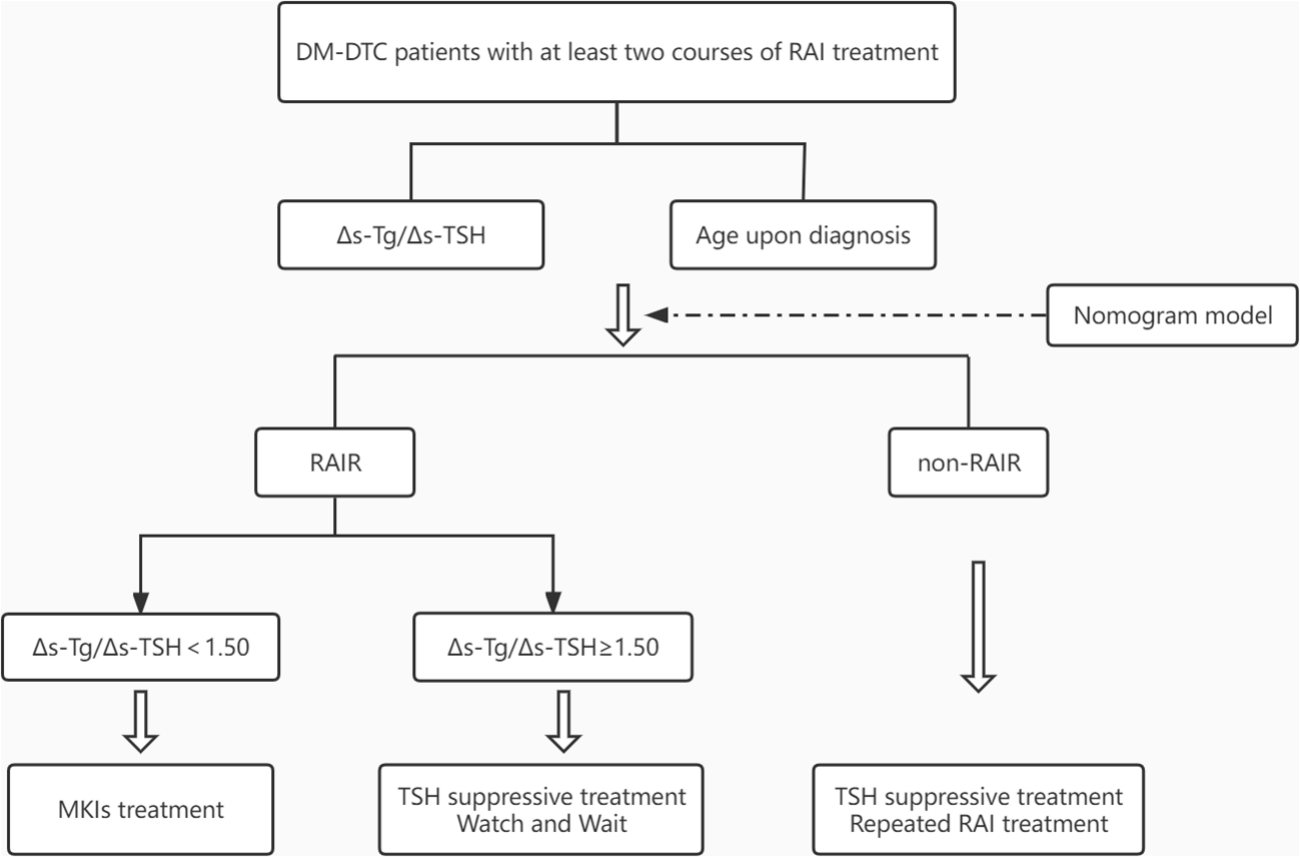
Flowchart for predicting RAIR and active surveillance of patients with DM-DTC. DM, distant metastases; DTC, differentiated thyroid cancer; TSH, thyroid stimulating hormone; MKIs, multi-kinase inhibitors.

Interestingly, when we further evaluated the biochemical progression using a 20% change of Tg among all the generally so-called RAIR patients defined by ATA criteria, differing biochemical progression can be identified as rapidly or slowly progressive patients with 1.50 as the Δs-Tg/Δs-TSH cut-off value. Patients with Δs-Tg/Δs-TSH <1.5 were apparently more progressive with a worse prognosis than those ≥1.50. The biochemical response of Tg has been reported to reflect the general tumor burden and progression, and is associated with structural response or relapse assessed by RECIST criteria (23, 30–32). The classification of RAIR has long been argued with controversy, as the four or five clinical scenarios may have different underlying genetic backgrounds and clinical progressions but were dealt with solid arbitrary management (33, 34). With the index derived from our study, different clinical progression might be further identified, which may shed light on the personalized management in RAIR patients, and even be helpful to optimize the currently watchful waiting strategy, and to tailor the timing of initiating systemic therapy such as MKIs from a clinical perspective. A workable flowchart was also suggested in this study to guide the active surveillance among RAIR patients (**Figure 6**).

Although BRAF^V600E^ mutation has been shown to be associated with RAIR (12, 35, 36), it was not manifested in the present study. We suppose the local genetic features reflected by the primary or regional tumor sites, or the heterogeneity between which might not thoroughly reflect the whole-body tumor genetic background, particularly the inaccessibility of distant metastasis due to ethic concern, while Tg, as a circulating marker, could comprehensively represent the overall biochemical tumor burden.

Our study is inevitably limited by its retrospective and single-center nature. Studies with larger sample sizes as well as multi-centers validation will be conducted subsequently.

In conclusion, we developed a convenient nomogram model integrating with Tg and age, which showed good predictive power for RAIR. By utilizing 1.50 as the cut-off value for Δs-Tg/Δs-TSH, the progression of RAIR may be further classified as a rapidly or slowly progressing group.

## Data availability statement

The raw data supporting the conclusions of this article will be made available by the authors, without undue reservation.

## Author contributions

JL and YL conceived and designed the study. CM and JS were the major contributors in performing the analysis, writing the manuscript, and preparing the figures and tables with support of WL. CM, JS, ZM, and YS collected samples and clinical parameters. JL and YL participated in the study design and edited the manuscript. All authors contributed to the article and approved the submitted version.
