# Supplementary material for: A user-friendly nomogram for predicting radioiodine refractory differentiated thyroid cancer
Source: Front Endocrinol (Lausanne). 2023 Feb 10;14:1109439. doi: 10.3389/fendo.2023.1109439 (PMC9950494; doi:10.3389/fendo.2023.1109439)
Supplement: Supplementary file 1 [file DataSheet_1.docx]

PredictABEL_1.2-4

tidyr_1.1.4

dplyr_1.1.0

gtsummary_1.7.0

dcurves_0.4.0

MASS_7.3-54

nomogramEx_3.0

pROC_1.18.0

caret_6.0-89

rms_6.2-0

SparseM_1.81

Hmisc_4.6-0

ggplot2_3.3.5

Formula_1.2-4

survival_3.2-11

lattice_0.20-44
